# Progress of Diseases of the Throat and Nose

**Published:** 1903-08-08

**Authors:** 


*(To be continued.)*

### Progress of Diseases of the Throat and Nose.

Adenoids are somewhat difficult to classify, but Marsh[1] thinks the following divisions might be made: (1) a central form comprising ($\alpha$) the ordinary form concentrically arranged with little or no lateral hypertrophy, and ($\beta$) a central boss or pad; (2) a lateral form with hypertrophy of the lateral bands most marked and often extending into the fossa of Rosenmüller; (3) a diffuse form with a general and irregular hypertrophy of all the lymphoid tissues in the nasopharynx and pharynx. He also classifies the symptoms into three groups—(1) obstructive, (2) catarrhal, (3) reflex. Amongst the last named he cites instances of headache, cough, spasm of the glottis, asthma, and sneezing, but he throws doubt on adenoids being responsible for the more remote reflex symptoms such as convulsions, epilepsy, stammering, and enuresis. Neglected adenoids seriously affect the development of the oro-nasal region, the thorax and body generally. In older children, he points out, the upper margin of the posterior nares loses its ovoid character and becomes angular like an inverted V. An interesting point is that when the growth of the soft palate is impaired, and consequently it cannot be approximated to the posterior wall of the pharynx, the voice, even after the thorough removal of the adenoids, remains of a very nasal character and causes disappointment. The author does not agree with Guze of Amsterdam in ascribing the condition known as "aprosexia" to the interference with the lymphatic channels passing from the meninges to the nasal fossæ. He states there is no anatomical evidence in support of this view, and he considers that the deafness and nasal obstruction would be quite sufficient to cause the condition. The cases in which operation is considered necessary or advisable are (1) aural cases, such as deaf-mutism, catarrhal or suppurative middle-ear trouble, temporary deafness from defective middle-ear aëration; (2) distinct nasal obstruction impeding development; (3) frequent and chronic catarrhal attacks interfering with health and education; (4) cervical adenitis associated with adenoiditis; (5) reflex conditions unrelieved by other treatment, and for which there is no other apparent cause. As an anæsthetic for the operation on children a preference is still given to chloroform administered until the conjunctival reflex is abolished, at which stage the laryngeal reflex is said to be unaffected. The recumbent position, with the head and shoulders slightly raised on a low pillow, is preferred for the operation to the "head over the end of the table."

In giving hints as to the treatment of chronic naso-pharyngeal catarrh, Freudenthal[2] also expresses some original opinions as to its causation. He describes the present mode of living as a return to "cave-dwelling." "In a 10 by 15 room the average human being passes 23 of the 24 hours of the day: the room is nothing but a cave-dwelling with the exception that it is tightly closed from all sides during the greater part of the year." If we speak of climatic influence in the production of disease, it is the climate of rooms which has to be considered. The writer claims that it is the absence of humidity from the air we breathe, and not its excess, which causes naso-pharyngeal catarrh. The atmosphere of a room should contain between 50 and 60 per cent. relative humidity, the minimum should never be below 40 per cent. In addition to other causes, he contends that the chief method of reducing the percentage of relative humidity is the mode of heating rooms, which results in an inhalation of dry air. Now the function of the noso being to charge with moisture the inspired air, it follows that, if the air be abnormally dry, the mucous glands of the mucous membrane are over-taxed, and they go on giving off moisture until "there is no more moisture to be given off." At first this gives rise to a hyperæmia, then hypertrophy, and after a variably long period to atrophy, the latter eventually affecting the underlying bones. On these assumptions Freudenthal, by way of treatment, strongly recommends sea-bathing in the summer, except for the very old. Woakes' agrees with this. Those who do not derive the full benefit from the seaside are sent to the mountains. In the winter a moist mild atmosphere, whether by the seashore or inland, is advisable. Those who cannot get away from home should live with open windows, and use a steam atomiser for moistening the air of the room. To obtain the necessary humidity for absorption by the system, the patient is told to undress completely in the bath-room whilst the hot water is running in. Before the tub is half filled with the hot water, the whole atmosphere of the room is saturated with steam. "By adding cold water the temperature of the bath-room becomes tepid. After remaining in the bath about ten minutes the patient should stay in the bath-room another 10, 15, or 20 minutes, dressed or, better still, undressed." Swimming-baths are also highly spoken of. As for local treatment, the writer expresses himself very sceptical regarding the value of cauterising hypertrophied turbinals, as he has seen too many atrophic conditions follow the procedure. He believes in removing crusts with the post-nasal applicator and applying a suitable lotion. Tampons saturated in ichthyol (5, 10, or 25 per cent.), left in the nose 10 to 20 minutes, loosen the crusts, and the patient is then able, by blowing his nose, to clear them away. Carbonic-acid gas is also found of great assistance in dry catarrh. Internal massage and electric currents are very serviceable.

Mackie[3] differs from the above opinion that atrophic rhinitis is the sequela of a pre-existing hypertrophic catarrh; in his experience he has found this condition almost invariably associated with sinus or other intra-nasal suppuration, and this view is strengthened by the success which has followed the treatment of these conditions. He, in support of this theory, cites 20 cases of ozæna, in every one of which the ozæna was associated with some local suppuration, the treatment of which, in not a few instances, effected a cure, while others, some of which are still under treatment, are progressing in that direction. His theory does not suffer, in his opinion, because of the disease occurring in early life, and of the semi-developed condition of the bony cavities at that period. He bases his conclusions on the following observations: (1) In cases of ozæna there is invariably a history of purulent discharge from the nose or naso-pharynx in early life; (2) this discharge, free at first, lessens in amount, but never quite disappears till it merges into ozæna, forming crusts and becoming offensive; (3) pus is always present in ozæna, and enters into the formation of crusts; (4) the establishment of free drainage and the cure of purulent discharge has proved more successful in treatment than any other method. A point of some importance is that in all cases of ozæna the lower turbinal is involved in the atrophic process, whereas the atrophy becomes less marked as we ascend to the upper regions of the nasal fossæ. This suggests that the atrophy is due to the descending discharges.

[1] Birm. Med. Rev., Aug. 1902. [2] Med. Rec., Aug. 9, 1902. [3] Quar. Med. Jour., Aug. 1902.

